# Draft genome sequence of epiphytic *Bacillus safensis* strain Cap07D, isolated from *Chenopodium album* L. seeds

**DOI:** 10.1128/mra.01339-25

**Published:** 2026-04-20

**Authors:** Vladimir K. Chebotar, Veronika D. Antonova, Maksim R. Losev, Maria S. Gancheva, Dmitriy V. Kudryavtcev, Olga A. Bortsova, Elena P. Chizhevskaya, Veronika N. Pishchik

**Affiliations:** 1 All-Russia Research Institute for Agricultural Microbiology, Pushkin, Russia; 2 Peter the Great St. Petersburg Polytechnic University 65068, Saint Petersburg, Russia; 3 Saint Peterburg State University, Saint Petersburg, Russia; University of Strathclyde, Glasgow, United Kingdom

**Keywords:** epiphytic, *Bacillus safensis*, genome sequence, *Chenopodium album*, seeds

## Abstract

Here, we report the genome sequence of the epiphytic *Bacillus safensis* Cap07D, a bacterium isolated from *Chenopodium album* L. seeds. The raw sequencing data were obtained using the NextSeq 1000 technology (Illumina).

## ANNOUNCEMENT

*Bacillus safensis* L. is a Gram-positive, spore-forming bacterium that belongs to the *Bacillus pumilus* group (1). Members of this group are widely distributed in both marine and terrestrial environments, particularly in soil ecosystems, and are frequently associated with plants as part of their natural microbiota (1). Strain Cap07D was isolated from the seeds of *Chenopodium album* L. collected in 2024 in the Stavropol region of Russia (45.156812° N, 42.098796° E). A flask containing 100 mL of sterile phosphate buffer and 10 g of *C. album* seeds was placed on an ultrasonic bath (Bandelin; 50 Hz) for 10 min. Then, the phosphate buffer solution containing microorganisms washed off from the grains was serially diluted, and 0.1 mL of various dilutions was inoculated on Petri dishes containing LB (Luria Bertani, Sigma-Aldrich, USA) agar medium, and plates were incubated at 28°C for 3 days. A single emerging colony of strain Cap07D was transferred to Luria–Bertani (LB) medium and cultured overnight at 30°C.

Genomic DNA from the strain Cap07D was extracted using the Wizard Genomic DNA Purification Kit (Promega, USA). Library preparation was performed using the KAPA HyperPlus Kit (Roche, Switzerland). Sequencing was conducted on an Illumina NextSeq 1000 platform with the NextSeq 1000/2000 P1 Reagents Kit (300 cycles), yielding a total of 2,081,668 raw paired-end reads with an average read length of 150 bp.

Raw read quality was inspected using FastP v0.23.4 (2). Genome assembly was completed with SPAdes v3.15.5 (3). The closest reference genome, *Bacillus safensis* GCF_008244765.1 (97.6%ANI), was identified by FastANI v1.34 (4) using reference genomes from the NCBI database (5). The resulting assembly was scaffolded with RagTag v2.1.0 (6) using the reference genome GCF_008244765.1. Contigs shorter than 500 bp were removed using SeqKit (7). The final draft genome assembly comprised 3,702,580 bp distributed across nine scaffolds with an average read coverage of 84.326×. Assembly metrics evaluated with QUAST v5.3.0 (8) reported N50 = 3,693,458 bp, L50 = 1, and a GC content of 41.67%. Genome completeness assessed with BUSCO v5.2.2 (9) with the bacillales_odb10 lineage data set indicated 99.8% complete single-copy BUSCOs.

Annotation of the assembled genome with the NCBI Prokaryotic Genome Annotation Pipeline (PGAP) v6.10 (10) predicted 3,742 protein-coding genes. Secondary metabolite biosynthetic gene clusters were analyzed with antiSMASH v8.0 (11), revealing 14 clusters involved in various biosynthetic pathways. These included siderophore-associated clusters, such as those predicted to synthesize bacillibactin and schizokinen, which generate iron-chelating metabolites crucial for iron acquisition and often linked to antimicrobial activity (12). Additional clusters related to biosurfactant and antimicrobial compound production, including lichenysin and fengycin, were also identified (13). Unless otherwise noted, default parameters were used for all software.

## Data Availability

This project has been deposited at GenBank under bioproject number PRJNA1242717. Whole genome assembly was deposited under accession number GCF_054073805.1. The raw reads were deposited in the Sequence Read Archive under accession number SRR32881993. antiSMASH annotation is available on Figshare (https://doi.org/10.6084/m9.figshare.29038718).
